# Diffuse neurofibroma of the chest and abdominal wall invading the diaphragm leads to diaphragmatic eventration: case report

**DOI:** 10.1186/s12893-021-01258-4

**Published:** 2021-05-24

**Authors:** Xian-shuai Li, Shu-qian He, Xian-guo Chen

**Affiliations:** Jinhua Hospital, Zhejiang University School of Medicine, 7 / F, Building 8, No. 365, Renmin East Road, Jinhua, Zhejiang China

**Keywords:** Diffuse neurofibroma, Diaphragm, Diaphragmatic eventration, Chest wall tumor, Case report

## Abstract

**Background:**

Neurofibromatosis type 1 (NF1) is an autosomal dominant condition with a high rate of new mutation and variable expression. Diffuse neurofibroma of the epidermis invading deeper organs is rare.We report a case of diffuse subcutaneous neurofibroma in the thoracoabdominal wall which had invaded the diaphragm and caused diaphragmatic eventration.

**Case presentation:**

We describe a patient with diffuse neurofibroma of the chest and abdomen who was admitted to the hospital due to sudden abdominal pain and a possible diaphragmatic hernia. We performed thoracotomy and found that the neurofibroma had invaded the diaphragm and caused diaphragmatic eventration.

**Conclusions:**

This occurrence has not been reported, and it shows that although neurofibromatosis is a benign disease, it still has the biological behavior of a malignant tumor and may cause a serious impact on and damage to other organs.

## Background

Neurofibromatosis type 1 (NF1) is an autosomal dominant condition with a birth incidence of approximately 1:3000 [1]. NF1-associated nerve sheath tumors include externally visible circumscribed cutaneous and subcutaneous neurofibromas that vary from few to innumerable and can evolve into infiltrative diffuse masses of varying sizes, the latter of which are termed diffuse neurofibromas, and often contain Meissner corpuscles [2]. NF1 was first described by Frederich von Recklinghausen in 1882. In 1987, the formal diagnostic criteria were published by the National Institutes of Health (panel) [3]. Disease penetrance is virtually 100% in adults with NF1, although there is great phenotypic variability in neurofibroma disease burden among patients, even those carrying identical NF1 germline mutations. Cutaneous and subcutaneous neurofibromas generally start to appear in late childhood and can grow in size and number throughout adulthood [4]. We report a case of diffuse subcutaneous neurofibroma in the thoracoabdominal wall that had invaded the diaphragm and affected its function.

## Case presentation

**Fig. 1:**
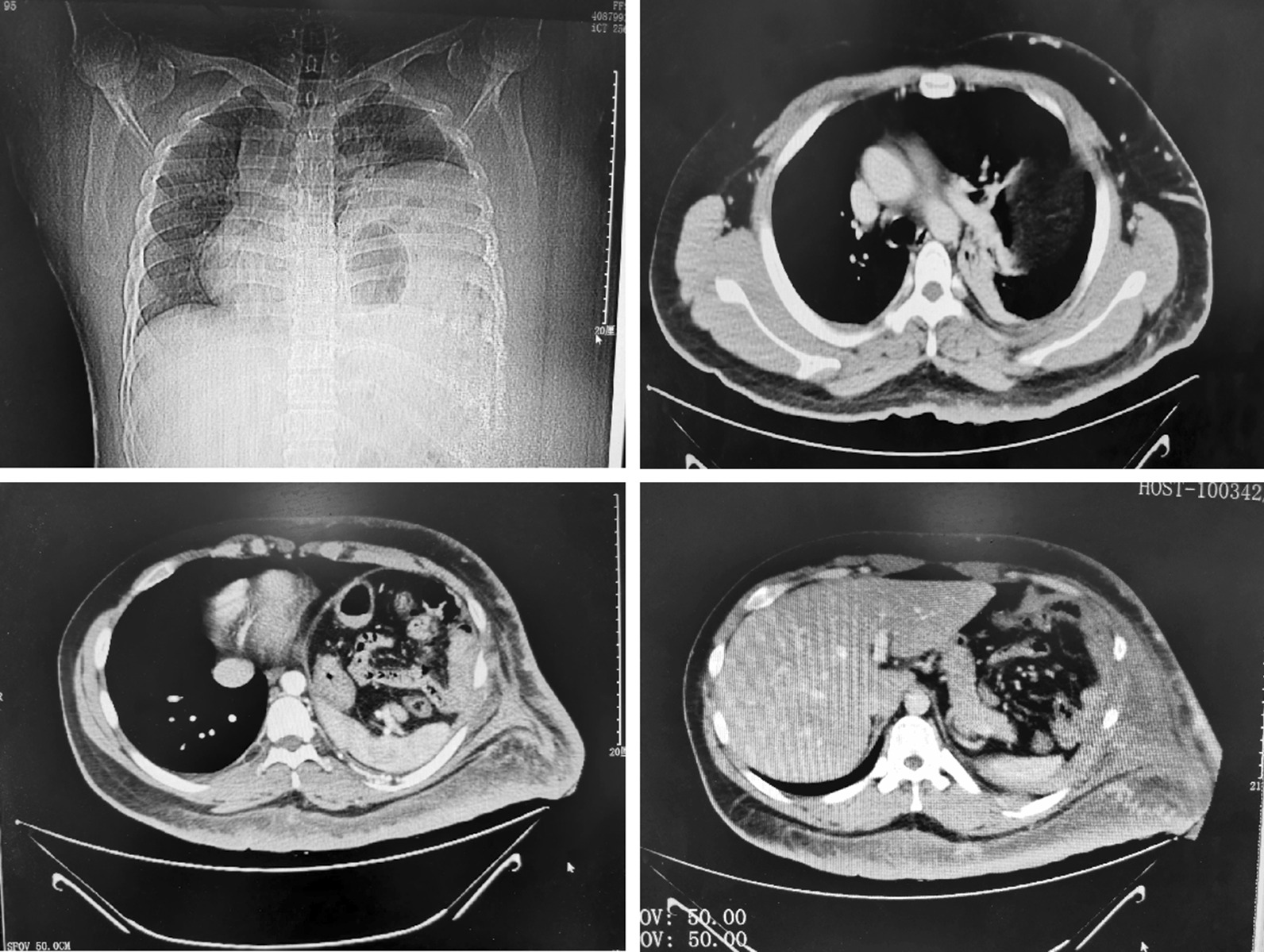
Chest X-ray and chest CT scan at admission

A 24-year-old male presented with diffuse skin lesions on the chest and abdominal walls from the age of 8, accompanied by pigmentation and epidermal nodular hyperplasia. Because of sudden spontaneous swelling and pain in the left upper abdomen that could not be relieved by rest or changing body position, he went to the emergency department of our hospital. The main vital signs of the patient were body temperature of 37.8℃, blood pressure of 140/93 mmHg, respiration of 24 beats/min, heart rate of 115 beats/min, and oxygen saturation of 96%. The emergency chest and abdominal enhanced CT scan showed the following: possible left diaphragmatic hernia; partially inflated left lung; massive left abdominal mass with hemorrhage, with the left 9th/11th ribs partially absorbed; left chest and back subcutaneous soft tissue thickened with an abnormal density; and bleeding nodules (shadow) on the chest and back skin (Fig. 1).

Physical examination showed that the breathing sound in the left lung was not clear, very large diffuse skin lesions with brown pigment spots in the left chest and abdomen, a large number of fibromatous hyperplasias on the epidermis, very large skin masses protruding outward, left abdominal compression pain (+), and abdominal muscle tension.

**Fig. 2:**
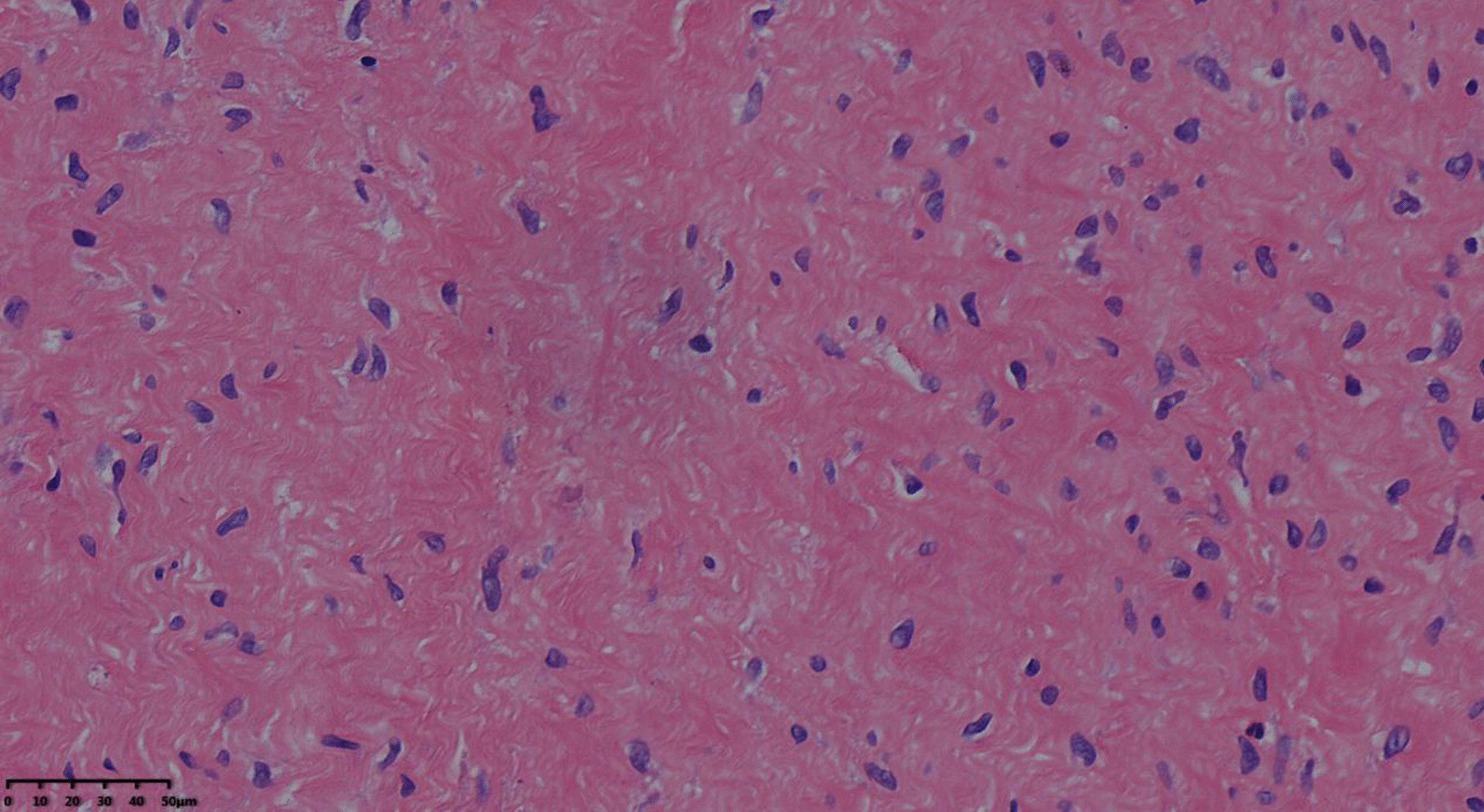
Pathology showed neurofibroma (40×)

Based on the patient's condition and CT findings, the possibility of a diaphragmatic hernia caused by spontaneous diaphragmatic rupture was not ruled out. After communicating with the patient's family members, we decided to perform thoracotomy. We selected the left 7th intercostal incision site, from the skin to the subcutaneous tissue there were a lot of neurofibroma tissue, brittle tissue and easy to bleed. The tumor covered a wide area, including the lateral chest wall, the back, and the whole abdomen. Because large areas of tumor tissue cannot be completely removed, only the thorax and abdominal problems can be dealt with. The left pleura and diaphragm were found to be invaded by tumor during the operation. The left diaphragm appears dark green with thinning tissue and reduced elasticity. The left diaphragm is significantly elevated, but not ruptured. Compression atelectasis was observed in the left lung. A small amount of bloody effusion was found in the abdominal cavity, and a portion of the bowel was embedded in the very large, twisted subcutaneous neurofibroma without necrosis. The bloody effusion was considered to be bloody exudation caused by intestinal cantun, and there is no obvious active bleeding in abdominal cavity. We found no obvious bleeding and necrosis in the abdominal cavity, and then a drainage tube was been placed. Diaphragmatic folding was performed. A portion of subcutaneous tissue and various diaphragmatic muscle tissues were removed during the operation and sent for rapid pathology. The diagnosis was neurofibroma (Fig. 2). The patient was admitted to the surgical ICU for care and treatment after surgery. The endotracheal intubation was removed 1 day after surgery, and the patient returned to the general ward 3 days after surgery. The patient had fever in the first 5 days, the highest temperature was 38.6 °C, other vital signs were stable, and then the temperature gradually dropped to normal. The postoperative abdominal pain disappeared and the incision healed well, and the patient was discharged 10 days after surgery.

## Discussion

"Neurofibromatoses" are a set of distinct genetic disorders that have in common the occurrence of tumors of the nerve sheath. They include NF1 and NF2 neurofibromas and schwannomas. NF1 affects multiple systems of the body. The major NF1-associated tumor is neurofibroma. In addition, clinical manifestations include bone dysplasia, learning disabilities, and an increased risk of malignancy [5]. Neurofibromas are the most prevalent benign peripheral nerve sheath tumors. Often appearing as a soft, skin-colored papule or small subcutaneous nodule, they arise from the endoneurium and the connective tissues of peripheral nerve sheaths [6]. There are three main types of neurofibromas: localized (the most common), diffuse, and plexiform. Approximately 20% of patients develop plexiform neurofibroma (PN), resulting in impaired quality of life. In the cases evaluated, 57.4% of diffuse PNs were located on the trunk, 19.2% on the head and neck, 12.8% on the lower limbs and 10.6% on the upper limbs [7]. In 1987, the formal diagnostic criteria were published by the National Institutes of Health (panel): (1) The maximum diameter of 6 or more milky coffee spots was more than 5 mm before puberty and more than 15 mm after puberty; (2) 2 or more neurofibromas of any type or 1 plexiform neurofibroma; (3) Brown freckles in armpit or groin; (4) Optic glioma; (5) Two or more Lisch nodules, namely iris hamartoma; (6) Obvious bone lesions: such as sphenoid dysplasia, long tubular bone cortex thin, with pseudojoint formation; (7) NF1 was confirmed in the first degree relatives. NF1 can be diagnosed if two or more of the above criteria are met. We found that the patient met the first three diagnostic criteria in physical examination.

Diffuse neurofibroma frequently grows as a plaque-like or infiltrative lesion involving the skin and subcutaneous tissues. Prominent internal vascularity is common. The mean patient age is 35.1 years [8]. Diffuse neurofibroma invading the spinal cord channel and soft tissues of the chest has been reported [9], but diffuse neurofibroma invading the diaphragm has not been reported. In our case, the patient was a young male. Diffuse neurofibromatosis had been progressing since childhood. Diffuse neurofibromatosis is characterized by the formation of extensive café au lait spots on the skin, nodular processes, diffuse growth of subcutaneous tumors, and continuous large-area tumor lesions on the chest, abdomen and back that have been unable to be resected. The neurofibroma of the patient was very extensive and extended deep from the chest and abdomen epidermis. A large number of tumor components were also observed in the subcutaneous tissue, reaching to the left pleura and adjacent left diaphragm. As a result, the left diaphragm appears dark green, thinner and less elastic than the normal diaphragm tissue at the right. Pathology of intraoperative left diaphragmatic tissue also confirmed that neurofibroma had invaded the diaphragm. Because only the invasion of the left diaphragm resulted in decreased muscle strength, the left half of the diaphragm developed diaphragmatic distention, and the preoperative chest radiograph also showed only elevation of the left diaphragm. Thus, the tumor is no longer limited to the skin and subcutaneous tissue, and further invasion into the diaphragm results in diaphragmatic lesions, contractility, increased toughness, further diaphragmatic swelling, upward compression of lung tissue, and abdominal organ displacement. The small intestine is embedded in the twisted tumor tissue channel, leading to acute abdominal pain. Due to the short interval between the onset of the patient's emergency and the operation, the possibility of the tumor invading the embedded intestinal tract was very small. In addition, there was no necrosis of the intestinal tract in the canal and no other abnormalities were detected during the operation, so the operation scope was not expanded. The surgical intervention described in the current case report, that is, diaphragmatic folding, makes abdominal organs return to their normal locations and relieves lung compression, but it is a symptomatic treatment that cannot prevent the further development of neurofibromatosis and cannot achieve radical treatment.

This case shows that although neurofibromatosis is a benign disease, it still has the biological behavior of a malignant tumor and may cause a serious impact on and damage to other organs. The surgical treatment of diffuse neurofibromatosis is limited, and early intervention (e.g., genetic engineering) may be a breakthrough in the future.

## Data Availability

All data generated or analysed during this study are included in this published article.

## Abbreviations

NF1: Neurofibromatosis type 1
NF2: Neurofibromatosis type 1
CT: Computerized tomography
PN: Plexiform neurofibroma
